# Larva and pupa of Megatoma (s. str.) undata (Linnaeus, 1758) with remarks on biology and economic importance (Coleoptera, Dermestidae)

**DOI:** 10.3897/zookeys.698.14049

**Published:** 2017-09-18

**Authors:** Marcin Kadej

**Affiliations:** 1 Department of Invertebrate Biology, Evolution and Conservation, Faculty of Biological Science, University of Wrocław, Przybyszewskiego 65, PL-51-148 Wrocław, Poland

**Keywords:** Exuvia, immature stage, seta, terga

## Abstract

An updated description of the final larval instar and pupa of Megatoma (s. str.) undata (Linnaeus, 1758) (Coleoptera: Dermestidae) is presented. Several morphological characteristics of *M. undata* larvae are documented and discussed: antenna, epipharynx, mandibula, maxilla, ligula with labial palpi, hastisetae, terga, and condition of the antecostal suture. The paper is fully illustrated and includes some important additions to extend notes on this species available in the references. Summarised data about biology, economic importance, and distribution of *M. undata* are also provided.

## Introduction

The genus *Megatoma* Herbst, 1791 is placed in the tribe Megatomini, subfamily Megatominae. Currently this group contains only 25 species (Kadej and Háva 2016 including one unrecognized species), and for this reason it is one of the smallest genera within the Dermestidae (= the Carpet Beetles; also known as skin or hide beetles). The genus is divided into four subgenera (Háva 2015). The nominal subgenus (*Megatoma* s. str.) includes five species, *Caucasotoma* Mroczkowski, 1967 one species, *Pseudohadrotoma* Kalík, 1951 10 species and *Perimegatoma* Horn, 1875 has eight species. Despite two existing revisionary studies of global *Megatoma* by Beal (1967) and Mroczkowski (1967), knowledge of the biology and ecology of the majority species is limited (Kadej and Háva 2016). The same can be stated for larvae. Only eight out of the 25 known species have any references in the literature in regards to larval morphology (Table 1). Moreover, most of the descriptions are inadequately short (Table 1).

**Table 1.** List of *Megatoma* species with references related to larval morphological characters.

Taxa	References	Available data
*Megatoma* Herbst, 1791	Lepesme and Paulian 1939	Short description in key (p. 167) [in French]
	Beal 1967	Short sentence in key (p. 290), brief description of larval morphology (p. 299)
	Zhantiev 1976	Short description in key (p. 94) [in Russian]
	Klausnitzer 1978	Short description in key (p. 167) [in German]
	Beal 1991	Short sentence in key (p. 439)
	Peacock 1993	Short description in key (p. 37)
	Klausnitzer 2001	Short description in key (p. 33) [in German]
Megatoma (Perimegatoma) ampla (Casey, 1900)	Beal 1967	Brief description of larval morphology (p. 306)
Megatoma (Perimegatoma) giffardi (Blaisdell, 1927)	Kiselyova and McHugh 2006	Illustration of mandible (p. 480), hypopharynx (p. 488), maxillae (p. 485), abdominal segments IV-IX (p. 492) and data matrix with larval characters (p. 498)
Megatoma (s. str.) ruficornis Aubé, 1866	Nonveiller 1959	Brief description of larval morphology [in French], illustration of larval habitus (p. 36, pl. I), pupa (p. 36, pl. I), hastiseta (p. 37, pl. II), spiciseta (p. 37, pl. II), tergites (p. 37, pl. II), feeding larvae (p. 38, pl. III) [description and illustrations have been made for *Megatoma pici* Kalík, 1952a which currently according to Háva (2015) is synonym of *M. ruficornis*]
Megatoma (s. str.) undata (Linnaeus, 1758)	Rey 1887	Description of larval morphology (p. 191-193) [in French]
	Lepesme and Paulian 1939	Illustrations of proleg, maxilla, labium, mandible (p. 163)
	Korschefsky 1944	Short description in key (p. 148) [in German], illustration of larval habitus (p. 154, pl. II)
	Hinton 1945	Short description in key (p. 258) and illustration of antenna (p. 256)
	Elbert 1976	Illustration apex (head) of hastiseta (p. 82)
	Zhantiev 1976	Short description in key (p. 99) [in Russian], illustration of apex (head) of hastiseta (p. 101)
	Elbert 1978	Illustration of apex of hastiseta (p. 110)
	Peacock 1993	Short description in key (p. 42) and on pages 42, 59, illustration of larval habitus – lateral view (p. 120), illustration of hastiseta from abdominal tergite VIII (p. 123), antenna (p.124), epipharynx (p. 127)
	Klausnitzer 2001	Illustration of larval habitus (p. 32, 34), antenna (p. 32), epipharynx (p. 32), hastiseta of abdominal segment VIII (p. 32)
	O’Toole 2010	Illustration of larval habitus (p. 68, 69, 70), exuvium (p. 71)
Megatoma (Pseudohadrotoma) variegata (Horn, 1875)	Beal 1967	Brief description of larval morphology (p. 301)
Megatoma (Pseudohadrotoma) conspersa Solsky, 1876	Zhantiev 1976	Short description in key (p. 99) [in Russian], illustration of apex (head) of hastiseta (p. 101)
Megatoma (Pseudohadrotoma) graeseri (Reitter, 1887)	Zhantiev 1976	Short description in key (p. 99) [in Russian]
Megatoma (Pseudohadrotoma) kaliki (Beal, 1967)	Beal 1967	Detailed description of larval morphology (p. 294); illustration of epipharynx (p. 292, 296)

According to the results of a phylogenetic analysis based on larval characters, *Megatoma* Herbst, 1792 is closely related to the *Trogoderma*-like Megatomini (such as *Reesa* (Milliron, 1939), *Cryptorhopalum* Guérin-Méneville, 1838, *Orphinus* Motschulsky, 1858, *Thaumaglossa* Redtenbacher, 1867, and *Trogoderma* Dejean, 1821) that are characterized by progressive desclerotization of the posterior portions of some abdominal terga (Beal 1967, Kiselyova and McHugh 2006). However, *Megatoma* seems to be most similar to *Trogoderma* and *Reesa*. The feature that distinguishes these three genera from *Cryptorhopalum*, *Orphinus* and *Thaumaglossa* is that all hastisetae and hastisetal brushes are inserted into sclerotized areas of terga, and never on membranes behind terga (= hastisetae that are concentrated on the lateral portions of the posterior abdominal terga, behind the row of stout spicisetae). In comparison, in genera such as *Cryptorhopalum*, *Orphinus*, and *Thaumaglossa*, brushes of the hastisetae are inserted on each side of a membrane behind the tergum (Beal 1991, Kiselyova and McHugh 2006).

Morphological characteristics of *Megatoma* larvae are given in the keys by Lepesme and Paulian (1939), Zhantiev (1976), Klausnitzer (1978, 2001), Beal (1967, 1991) and Peacock (1993). The larval features that distinguish *Megatoma* from related genera *Reesa* and *Trogoderma* were given by Peacock (1993) and Beal (1991), and are mainly expressed by the length of setae of the tarsungulus (pretarsus), number and morphology of the middle four setae of the labor-epipharyngeal margin as well as morphology of the tergites. In regards to setae of the tarsungulus, in *Megatoma* they are equal in length, while in *Trogoderma* unequal, and in *Reesa* subequal. The setae of the labor-epipharyngeal margin consists of two broad inner and two broad (spatulate) outer setae in *Megatoma*; while two broad inner and two narrow outer setae are detected in *Trogoderma* and *Reesa*.

A further difference between *Megatoma* and *Trogoderma* and *Reesa* is seen in the location of dense brushes (= tufts) of hastisetae on the abdominal terga. In *Megatoma* they are located on abdominal segments VI–VIII, while in *Trogoderma* the brushes are mostly on abdominal terga V–VIII (longest and thickest on VI–VIII), while in *Reesa* they are situated on abdominal terga I–VIII (but the longest and thickest are on VI–VIII).

According to Peacock (1993), *Megatoma* also differs from *Trogoderma* and *Reesa* in the length of antennomere II, which is twice as long as antennomere III in *Megatoma*, while in *Trogoderma* and *Reesa* it is not more than half as long.

The larvae of *Megatoma* are also similar to those of *Anthrenus* Geoffroy, 1762 and *Ctesias* (Fabricius, 1792). The main difference between these genera is a set of dense brushes of hastisetae on some of the abdominal terga. In *Anthrenus* and *Ctesias* they are located on the membrane behind the tergum, in *Anthrenus* on each side of abdominal terga V–VII, while in *Ctesias* on each side of abdominal terga IV–VII. In contrast, in *Megatoma* the brushes of hastisetae are inserted on the sclerotized area (never on membranes behind terga), and particularly on abdominal terga VI–VIII (Beal 1967). Additionally the body of an *Anthrenus* larva is broadest at abdominal segments IV–VI (Beal 1991), or according to Peacock (1993), II–V.

Current work is a continuation of previous articles devoted to the morphology of immature stages of Dermestidae (Beal and Kadej 2008, Kadej 2012a, b, c, Kadej and Jaroszewicz 2013, Kadej et al. 2013a, b, Kadej and Guziak 2017, Kadej and Guziak “in press”, Kadej et al. 2017). In this paper, an updated description of the larva of *Megatoma undata* (Linnaeus, 1758) is given. This species represents the nominal subgenus Megatoma s. str. and is widely distributed in Europe.

*Megatoma undata* is associated with woodland habitats. The species has been mainly observed in forests, under the bark of old trees, inside of hollows, in corridors of other insects in dead wood (e.g. beetle borings), in the nests of Aculeata (e.g. solitary bees), in old bee-hives or even inside of bird boxes (Burakowski et al. 1986, Peacock 1993, Kadej 2005, Byk et al. 2006, Takano et al. 2012).

There are few papers that correspond to larval morphology of *M. undata*. However, as shown in Table 1, most of them are limited to only a few sentences in the key or brief description with few schematic illustrations; extended notes for this species available in the references are presented. The following set of larval characters are described, illustrated, and discussed for *M. undata* for the first time: pronotum, abdominal segment I, VII–IX, and the frons. The pupal stage is also described and illustrated for the first time. Summarized data about the biology and economic importance of *M. undata* are also provided.

## Materials and methods

For morphological examination, larvae or exuvia of the last-stage were studied using specimens stored in ethanol. The studied material came from the collection of the Department of Invertebrate Biology, Evolution and Conservation, University of Wrocław (DIBEC). Larvae/exuvia were boiled for 3-10 minutes in 10% solution of KOH, and then rinsed with distilled water. Then morphological structures were placed in distilled water for ~1 hour for the purpose of cleaning and softening the material. All structures were put into glycerine on slides. The morphological structures were examined under a Nikon Eclipse E 600 phase contrast microscope with a drawing tube attached, and a Nikon SMZ-800 binocular microscope; the samples were mounted in glycerine and viewed with transmitted light. Photos were taken with Canon 500D and Nikon Coolpix 4500 camera under Nikon Eclipse 80i or Nikon SMZ-800. Apart from the written description, plates with the drawings of selected elements have also been prepared for the larva. The terminology used in this paper follows Kiselyova and McHugh (2006).

**Figure abbreviations are as follows**:

**ac** acrotergite;

**as** antecostal suture (ridge);

**br** transverse row of placoid sensillae on epipharynx;

**cs** camapniform sensilla;

**dst** distal epipharyngeal sensillae;

**dmr** dorsomesal row of setae on lacinia;

**er** epipharyngeal rods;

**g** galea;

**l** lacinia;

**lp** labial palp(i);

**mp** mesal pair of labor-epipharyngeal setae;

**msr** mesal row of setae on lacinia;

**mxp** maxillary palp(i);

**p2** second pair of labor-epipharyngeal setae;

**pls** placoid sensilla;

**prst** prostheca;

**s** sensorium (accessory sensory papillae);

**sbp** subproximal epipharyngeal sensillae.

All materials are in **DIBEC**.

## Taxonomy

### Subfamily Megatominae Leach, 1815

#### Tribe Megatomini Ganglbauer, 1904

##### Genus Megatoma

Taxon classificationAnimaliaColeopteraDermestidae

Herbst, 1792

Figs 1–3 , 4–6 , 7–16 , 17–21

Megatoma (s. str.) undata (Linnaeus, 1758)

###### Material examined.

One larva, and 15 exuviae. Original label: “Kazimierz n/W 16.10.1950 leg. M. Mroczkowski [Kazimierz under Vistula]”; seven larvae ”Polonia Kazimierz n/W, 28.3.1951, cult. M. Mroczkowski [Kazimierz under Vistula] / larwy 16.10.1950 w komorach samotnych pszczół w ścianie lessowej [larvae in the chambers of solitary bees in the wall of loess], leg. M. Mroczkowski”. Seven larvae. Original label: ”Kazimierz n. Wisłą w gniazdach błonkówek [Kazimierz under Vistula in the nests of Hymenoptera] 17.V.1955, leg. M. Mroczkowski”. Seven exuviae. Original label: “Kazimierz n. Wisłą w gniazdach błonkówek [Kazimierz under Vistula in the nests of Hymenoptera] 27.V.1955, leg. M. Mroczkowski”. One larva, three exuviae, three pupae. Original label: “Kazimierz nad Wisłą [Kazimierz under Vistula] 16.VIII. 1955 ex larva, leg. et cult. M. Mroczkowski”.

###### Description.

**Figures 1–3.**
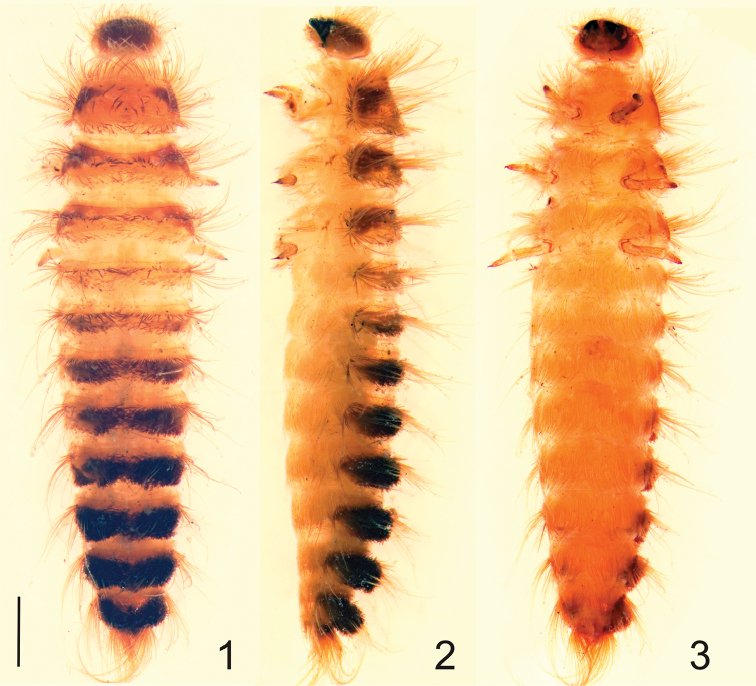
Mature larva of Megatoma (s. str.) undata (Linnaeus, 1758). **1** Dorsal view **2** Lateral view **3** Ventral view. Scale bar 0.1 mm.

Larva, last instar. Body length 5.0–11.0 mm. Body fusiform, relatively long, flattened, not hunchbacked (Figs 1–3). Integument of head, nota, and terga brown. Head darker than terga. Tergal plates sclerotized (Figs 1–2), sterna hyaline and unpigmented (Fig. 3), femora and tibiae light yellowish (Figs 2–3). Thoracic terga I–III with distinctly dark brown patches at sides (Figs 1–3), sometimes extending to middle on terga II and III. Setae (spicisetae and hastisetae) on tegra and sterna brown (Figs 1–3). Head of hastisetae short; 3–4 times long as wide (Fig. 8). Head protracted and hypognathous (Figs 2, 3). Stemmata (probably 5) present on the head, arranged in two semi-oblique rows. Frons triangular (Fig. 9), without frontal, median tubercule; covered with spicisetae and nudisetae (the latter present only at the anterolateral angles of the frons); setal patterns as on Fig. 9. Antennae orientated anterolaterally (Fig. 1); composed of three antennomeres (Fig. 7). Terminal antennomere 3.0 times as long as wide, with two small sensory sensilla (appendages): one on the apex, second one under the apex; and two campaniform sensillae under half of length of antennomere (near base). Ratio of length of terminal antennomere to length of penultimate and antepenultimate antennomeres combined nearly 0.4:1.0. Sensorium in ventral position, below the apex of antennomere II. Single seta present on antennomere II near apex (opposite to sensorium). Three campaniform sensillae present on antennomere II – two under sensorium and one close to the base of the segment. Antennomere I with 3–7 long setae (probably there are not any campaniform sensillae (cs) (Fig. 7)). Gula separate from postmentum; epicranial stem present. Median endocarina absent. Labro-epipharyngeal margin with 12-14 setae in the outer series. Mesal (mp) of labro-epipharyngeal setae and second pair (p2) broad and spatulate. On ventral side of epipharynx distal epipharyngeal sensillae (dst) arranged in one group of 6 in two rows (four in the upper one, and two below), but not encircled by distinct furrow (they are loosely grouped in faintly defined fusiform callosity, Fig. 12). Four to six sensory cups in the subproximal epipharyngeal sensilla (sbp) are present. Middle pair larger and lateral sensilla smaller, directed down to the basal transverse row (br) of placoid sensillae. Epipharynx with nine sensory cups in the proximal transverse series (br). Epipharyngeal rods (er) present and diverging proximally. Lateral setae on epipharynx absent (Fig. 12). Dorsal surface of labro-epipharynx with many setae. Mandible brown with dark brown (almost black) apices; apical teeth and ventral accessory process absent. Apical half of mandible heavily sclerotized and sharply delineated from the basal half (Figs 10, 11). Mandibular mola and pseudomola absent. Hyaline lobe at ventral base of mandible absent. Prostheca falciform (Fig. 10 and 11), brush of setae absent mesally near the mandibular base. Placoid sensillae (pls) present in basal part (in approximately 1/4 of the dorso-lateral length) of mandible (Fig. 10). Maxillary palp composed of three palpomeres with terminal palpomere longest. Ratio of terminal palpomere length to the two proceeding palpomeres combined 1.3:1.0. First palpomere with two seta (on the Fig. 14 one of them is lacking), second palpomere with 3 setae (on the Fig. 14 two of them are lacking), and third palpomere with two campaniform sensillae and group of 5–6 small sensillae situated in the apical area (Fig. 14). Lacinia with two, heavy sclerotized lacinial teeth, straight at apex. Sclerotization of lacinia separated from stipes. Seven to as many as thirteen straight slender to thick setae present in a dorsomesal row on lacinia (dmr) (Fig. 13). Mesal row of setae on lacinia (msr) composed of one basally thickened seta (Fig. 14). Galea arising from stipes terminates close to the apex of lacinia. The apical area of galea covered densely with setae. Stipes with 14–18 long setae placed mainly near the anterio-lateral margin, two setae present near the inner margin (close to the first palpomere) (Fig. 14). Hypopharynx hyaline. Bridge sclerite (central part of the distal element of the hypopharyngeal sclerome) appearing jointed medially. Anterior arms of bridge sclerite and distal lateral sclerites of hypopharynx absent. Ligula with 13–15 lanceolate setae (Figs 15 and 16). Labial palp with two palpomeres (Fig. 15). First segment wider than second segment; without setae on the disc. Terminal labial palpomere 2.0 times as long as wide, with group of 7–8 small sensillae in the apical area and two campaniform sensilla (cs) (Fig. 14).

Antecostal suture smooth and distinct, present on nota I–III and abdominal terga I–VIII. Acrotergites of notum I without setae (Fig. 17), while acrotergites of nota II–III and abdominal terga I–IX(?) with setae (Figs 18–21). Notum I with long, stout, large spicisetae along anterior (here directed anteriorly under the head) and lateral margin; only few spicisetae located along posterior margin (here directed latero-posteriorly and vertically - upright). The setae on the posterior margin are situated near the latero-posterior angle, with some additionally near the posterior suture, and some also present on central area of disc of notum I (Fig. 17). Nota II, III and all abdominal terga with median row of large spicisetae, and along lateral margins of terga (Figs 18, 20–21). They are mainly directed latero-posteriorly and vertically (upright). Hastisetae present both on nota I–III as well as on abdominal terga I–VIII, forming dense lateral brushes (= tufts) on abdominal terga, but the longest and the thickest are on segments (V)VI–VIII (the aggregation of hastisetae arises even from tergum IV and they become significantly denser and thicker closer to the posterior end of the body). Setal patterns of abdominal tergum I with numerous large spicisetae in median row and along lateral margin; posterior margin (under the median line of spicisetae) bearing mainly hastisetae (Fig. 18). Abdominal tergum VII as illustrated (Fig. 20). Abdominal tergum VIII without pair of abdominal pits (oval apertures); setal patterns as illustrated (Fig. 21). Abdominal tergum IX reduced with numerous long spicisetae (Fig. 19). Legs (tibia, femur and trochanter) covered with many lanceolate setae. Claws dark brown. Ratio of tibial to femoral length 0.8:1.0. Pretarsus with two narrow lanceolate setae inserted at base. Length of posterior pretarsal seta equal to anterior pretarsal seta.

**Figures 4–6.**
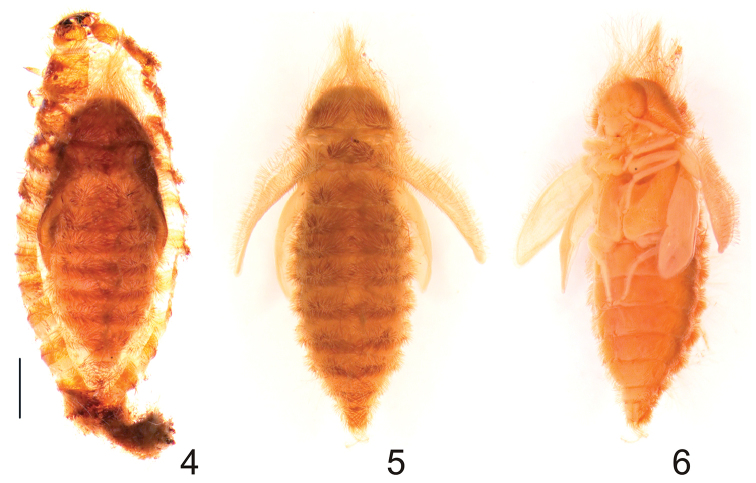
Pupa of Megatoma (s. str.) undata (Linnaeus, 1758). **4** Dorsal view (pupa inside of the last larval skin) **5** Dorsal view **6** Latero-ventral view. Scale bar 0.1 mm.

Pupa: length 5.0–7.0 mm (Figs 4–6). Integument yellowish brown with erect, brown coloured spicisetae distributed rather uniformly on head, dorsum, and wings. The longest spicisetae present on head (Figs 4–6). Gin traps and urogomphi absent (Figs 4–5). Pupa remains within the last exuvia (= larval skin, Fig. 4). It is anchored by two clusters of long fine setae inserted on each side of the abdominal tergum VIII.

###### Distribution.

Widely distributed in Europe. The species has been also recorded from the Caucasus (Háva 2015).

###### Biology.

Adults are seen from April to October (in Poland) and can have two generations per year. The species overwinters as either an adult or larva. Individuals can be found on the bark of trees, close to places with leaking sap, under bark (including that of elm, larch, oak, crab apple, sycamore, willow, maple, ash, and beech trees), in spider webs, inside bird nests or boxes, bee hives, or on the walls of old timber-built houses or barns (Mroczkowski 1975, Peacock 1993, Kadej 2005, Byk et al. 2006).

The immature stages have been found in nests of different Aculeata (where they feed on both their food and exuviae and pupae). Brechtel (1986) and O´Toole (2010) recorded the larva of *M. undata* from nests of *Osmia rufa* (Linnaeus, 1758), while Rey (1887) described it from galleries of saproxylic bees *Xylocopa violacea* (Linnaeus, 1758). It has also been recorded as a predator of the pupae of the moth *Lymantria dispar* (Linnaeus, 1758) in oak forests (Mihalache et al. 1995). According to observations of O´Toole (2010) larvae feed on larval exuviae of the red mason bee, their fecal pellets, on the silk cocoons spun by the pre-pupal bees as well as on dead adults. In buildings, larvae feed on products of animal origin such as dry insect specimens in collections, skins, furs, and old wool (Fowler 1889, Joy 1932, Takano et al. 2012).

Unlike the larvae, adults feed on pollen (Mroczkowski 1975, Peacock 1993). Beetles were most often found in their breeding sites, and rarely on flowers. Hunter (1959) reported both adults and larva from larval burrows of cerambycids such as *Molorchus minor* (Linnaeus, 1758), *Tetropium gabrieli* Weise, 1905 and *Anaglyptus mysticus* (Linnaeus, 1758). Allen (1958) observed *M. undata* associated with the spider *Salticus scenicus* (Clerck, 1757).

###### Economic importance.

In this regard, this species has low importance because it has never been recorded on a mass scale and occurs mostly in natural conditions (Mroczkowski 1975). It is generally an “outdoor” species that occasionally enters houses to feed on products of animal origin. Thus, the species has not been classified as a typical pest (Takano et al. 2012). Moreover, some authors have even classified this species as a saproxylic beetle (Byk et al. 2006, O´Toole 2010) or a woodland indicator (Garland 1983).

**Figures 7–16.**
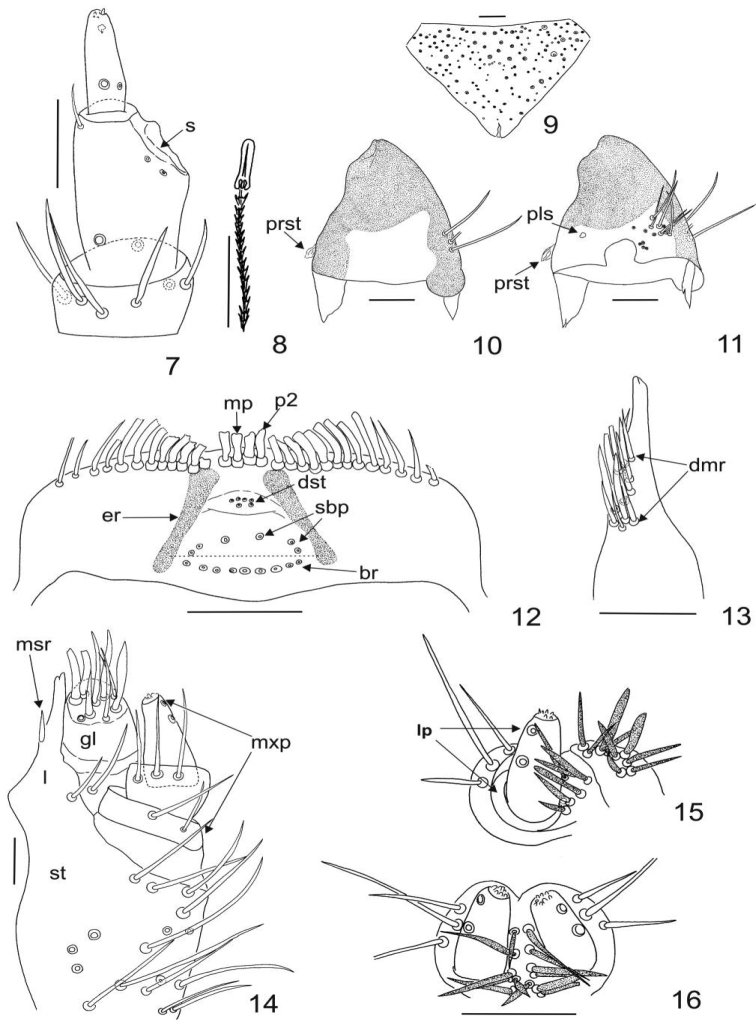
Mature larva of Megatoma (s. str.) undata (Linnaeus, 1758). **7** Antenna (dorso-fronto-lateral) **8** Head (apex) of hastiseta **9** Frons (dorsal) **10** Mandibula (dorsolateral) **11** Mandibula (dorsal) **12** Epipharynx (ventral) **13** Lacinia **14** Maxilla **15** Labium with labial palp (frontolateral) **16** Labium with labial palpi (frontal). Scale bar 0.1 mm.

**Figures 17–21.**
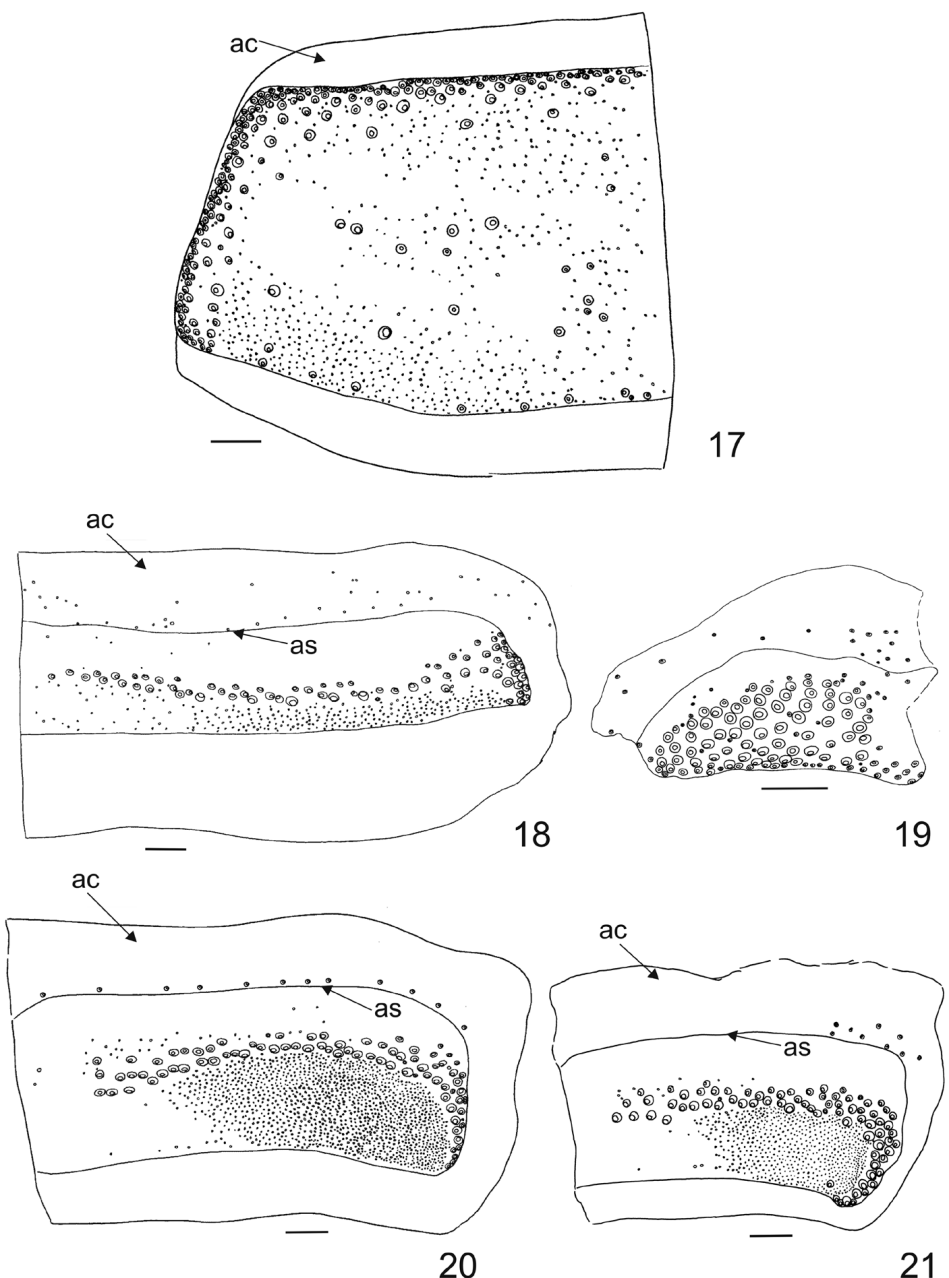
Mature larva of *Megatoma* (*s. str.*) *undata* (Linnaeus, 1758). **17** Pronotum (dorsal, left half; circles with central ring represents points of insertion of spicisetae, small circles represents points of insertion of hastisetae) **18** Abdominal tergum I (dorsal, right half; circles with central ring represents points of insertion of spicisetae, small circles represents points of insertion of hastisetae) **19** Abdominal tergum IX (dorsal, circles with central ring represents points of insertion of spicisetae) **20** Abdominal tergum VII (dorsal, right half; circles with central ring represents points of insertion of spicisetae, small circles represents points of insertion of hastisetae) **21** Abdominal tergum VIII (dorsal, right half; circles with central ring represents points of insertion of spicisetae, small circles represents points of insertion of hastisetae). Scale bar 0.1 mm.

## Discussion

There are only eight references to larval morphology for the genus *Megatoma* Herbst, 1792 (see Table 1). Only two species out of the eight represent the nominal subgenus, while others belong either to the subgenus Perimegatoma Horn, 1875 (two species) or to the subgenus Pseudohadrotoma Kalík, 1951 (four species) (Háva 2015). Beal (1967) suggested a set of characters that probably can enable larval distinction between the subgenera *Pseudohadrotoma* and *Megatoma* as follow: 1) row of spicisetae along posterior margin of each abdominal tergite in addition to the median raw of large spicisetae; 2) the anterior abdominal tergites bear some small spicisetae on the disc behind the median row of large spicisetae; 3) the abdominal terga VIII lack an antecostal suturae. I was not able to study any of the species of subgenus Pseudohadrotoma and therefore can present no evaluation.

Among *Megatoma*, two species out of four with larval references (M. (Perimegatoma) ampla (Casey, 1900) and M. (P.) giffardi (Blaisdell, 1927)) have been recorded from the Nearctic Region (Háva 2015). Two others, *M. undata* (Linnaeus, 1758) and *M. ruficornis* Aubé, 1866, are distributed in the Palaearctic Region (Háva 2015). Due to limited descriptions and fragmentary data, it is difficult to prepare a detailed differentiation for larva of abovementioned species beyond a superficial comparison. For instance M. (s. str.) undata can be distinguished from some other known species by pigmentation. The thoracic terga (nota I–III) have characteristic dark brown pigmentation (see above in the description) and abdominal segments are uniformly brownish yellow; in M. (Perimegatoma) ampla the anterior half of each tergum and notum are pigmented a medium brown except for notum I, which is a yellowish brown; in M. (P.) variegata the nota I–III as well as the anterior abdominal terga are darkly pigmented, except for a median yellowish line; in case of M. (P.) kaliki, medium-brown pigmentation is present on the dorsal surface (Beal 1967).

Moreover, some published data contradict each other. For example, the presence or absence of a single seta near apex of antennal segment II (compare with Fig. 7) is noteworthy. Additionally Beal (1967: 301) noted that mature larvae of M. (s. str.) undata (compare with Peacock 1993, 124), M. (Pseudohadrotoma) variegata (Horn, 1875) and M. (P.) cylindrica (Kirby, 1837) have no seta on antennal segment II. My observations contradict this thesis (compare with Fig. 7, current paper) as well as illustrations of the antenna of M. (s. str.) undata by Hinton (1945: 256) and Klausnitzer (2001: 32). The antennae of M. (s. str.) undata is much more similar to M. (s. str.) ampla Beal (1967: 301). This inconsistency is probably associated with availability of immature larva for the study. It is very likely that younger larval instars do not possess that seta on segment II or the above mentioned authors could not see the seta as it had been lost before the examination.

The same inaccuracy can be shown with the epipharynx. Contrary to the published data of Peacock (1993: 127) and Klausnitzer (2001: p. 32) the epipharynx of specimens studied by me exhibit some similarities with those of M. (Pseudohadrotoma) kaliki (Beal 1967) shown by Beal (1967: 292). The main differences are associated with numbers of sensillae in the row of subproximal epipharyngeal sensilla (sbp) and sensory cups in the proximal transverse series (br). In Peacock (1993: 127) there are eight sensory cups in the proximal transverse series (br), while my specimens have 9. Moreover, only two subproximal epipharyngeal sensilla (sbp) are present on the figure by Peacock (1993: 127) and Klausnitzer (2001: 32), while four to six were observed by me (compare with Fig. 12, current paper).

Lastly Rey (1887: 191) wrote that maxillary palps of *M. undata* consist of four segments. This observation is also not consistent with results of this study. Probably Rey improperly interpreted this feature, as *Megatoma* has only three segments like in other genera within the subfamily Megatominae.

These examples all show how much care must be paid during the study of immature stages of Dermestidae. Proper description requires both the attention of the researcher and well preserved material as well (and best if long series are available to show the range of variability). Focusing on larval stages can significantly support our current knowledge of taxonomy. Kiselyova and McHugh (2006) proved how useful immature stages are in studying phylogenetic relationship among genera and certainly the larval characteristics could also significantly support taxonomic reasoning within a particular genus.

The results of current study support the phylogenetic placement of *Megatoma* provided by Kiselyova and McHugh (2006). Most of the larval characteristics of Megatoma (s. str.) undata (Linnaeus, 1758) described herein overlap those included in their matrix for Megatoma (Perimegatoma) giffardi (Blaisdell, 1927) (Kiselyova and McHugh 2006: 498). It is especially interesting because M. (s. str.) undata represents the Palaearctic region, while Megatoma (P.) giffardi the Nearctic species of the genus *Megatoma*. As noticed by Kadej and Hava (2016), until 1945 most of the Nearctic species of *Megatoma* s. str. were placed in a separate genus *Perimegatoma* Horn, 1875 (Beal 1967, Mroczkowski 1967). Hinton (1945) synonymized *Perimegatoma* Horn, 1875 with *Megatoma* Herbst, 1791. According to Mroczkowski (1967) the “North American species” probably constitute a separate evolutionary line. If this is the case, then the recognizable morphological similarities between Palaearctic and Nearctic species would be the result of parallel evolutionary processes. The results of the present study, although supporting this hypothesis, need to be confirmed by a wider study of larvae of the other species.

## Supplementary Material

XML Treatment for Megatoma
